# Genome-wide analysis of Alfalfa AP2/ERF gene family and abiotic stress response

**DOI:** 10.3389/fpls.2026.1776336

**Published:** 2026-02-19

**Authors:** Rui Qiu, Jiani Wang, Wenqiang Fan, Yingtong Mu, Yutong Zhang, Lina Zhao, Fengling Shi

**Affiliations:** 1 Key Laboratory of Forage Cultivation, Processing and High Efficient Utilization, Ministry of Agriculture, Hohhot, China; 2 Key Laboratory of Grassland Resources, Ministry of Education, Hohhot, China; 3 College of Grassland Science, Inner Mongolia Agricultural University, Hohhot, China

**Keywords:** abiotic stresses, alfalfa, AP2/ERF transcription factor family, expression pattern, gene structure

## Abstract

**Introduction:**

AP2/ERF transcription factors represent one of the largest gene families in plants, playing key roles in regulating growth, development, and responses to abiotic stresses. Alfalfa (*Medicago sativa* L.) is an important leguminous forage crop, whose growth and yield are often severely constrained by abiotic stresses.

**Methods:**

In this study, we identified 110 MsAP2/ERF genes from the genome of the alfalfa cultivar ‘Xinjiang Daye’ through a genome-wide screening and mapped them onto 28 chromosomes. Gene duplication analysis revealed that this family has undergone extensive tandem duplication events and strong purifying selection during its evolution.

**Results:**

Collinearity analysis identified 104 and 33 putative orthologs between *Medicago sativa* and *Medicago truncatula* L. and *Arabidopsis thaliana*, respectively. These genes exhibit considerable diversity in gene structure and conserved protein motifs, and their promoter regions are enriched with various cis-acting elements related to abiotic stress responses. Based on publicly available transcriptome data, a total of 12 MsAP2/ERF genes were found to be differentially expressed under cold, salt, and drought stresses. Among them, several genes, including *MsERF45*, *MsERF54*, and *MsERF55*, showed significant responses to all three types of stress, as confirmed by RT-qPCR assays.

**Discussion:**

These findings provides valuable insights into the functions of MsAP2/ERF genes in stress resistance mechanisms in alfalfa to support future stress-tolerant alfalfa breeding efforts.

## Background

The AP2/ERF superfamily represents a highly conserved class of transcription factors in plants, characterized by the presence of one or more AP2/ERF domains composed of approximately 60–70 amino acids. This domain possesses specific DNA-binding capabilities, forming the basis for its transcriptional regulatory functions (Yamasaki et al., 2008). Jofuku et al (Jofuku et al., 1994). identified and cloned the AP2 gene, a key regulator of flower development, in *Arabidopsis thaliana*. The protein encoded by this gene contains two AP2/ERF domains. Subsequently, the Ohme-Takagi team (Ohme-Takagi and Shinshi, 1995) identified ethylene response factor-binding proteins in tobacco (*Nicotiana tabacum* L.) containing a conserved ERF structure, including ERF1-4. Faraji et al (Faraji et al., 2020). conducted a genome-wide identification of the AP2/ERF gene family in tetraploid wheat (*Triticum turgidum* ssp*. durum*) using RNA-seq. The study identified 271 AP2/ERF gene family members and revealed their potential functions in enhancing drought and salt tolerance in wheat, traits of significant agronomic importance for crop improvement. Many AP2/ERF family proteins are involved in the regulation of biological processes, metabolic regulation and abiotic stress response (Pillai et al., 2020). Proteins within the AP2/ERF family participate in a broad range of cellular processes, including the regulation of metabolism and response to abiotic stress (Zafar et al., 2024). The AP2/ERF transcription factor family plays a pivotal role in plant development and stress responses. Among these, the AP2 subfamily regulates developmental processes including embryogenesis, seed formation, and floral organ development (Meng et al., 2023), the DREB subfamily enhances abiotic stress tolerance by activating downstream target genes (Hu et al., 2020; Su et al., 2023; Wang et al., 2024), while RAV transcription factors modulate leaf physiology and stress adaptation (Liu et al., 2021). ERF proteins link hormonal signals (e.g., ethylene, ABA) to abiotic stress responses through their conserved AP2/ERF domain and GCC box binding functions. By constructing dynamic protein interaction networks, they integrate epigenetic and transcriptional regulation to establish a core adaptation framework, providing key targets for breeding stress-tolerant crops (Shinozaki et al., 2003; Jung et al., 2017).

Alfalfa (*Medicago sativa* L.), a perennial legume widely recognized as the ‘king of forage’, is one of the world’s most important forage crops due to its high nutritional quality, strong palatability, and abundant crude protein content (Suwignyo et al., 2023). Beyond its agronomic value, alfalfa also exhibits remarkable ecological benefits. It adapts effectively to marginal soils, developing an extensive root system characterized by a deep taproot, high tillering capacity, and dense ground cover, which contributes significantly to soil and water conservation (Xiangrong et al., 2008).

However, the growth and yield of alfalfa are often severely constrained by abiotic stresses such as drought, salinity, and low temperatures, which represent major limitations to global agricultural production. These stresses frequently interact synergistically with biotic stresses, amplifying their negative impacts on plant development and productivity (Hsieh et al., 2013; Dossa et al., 2016). To cope with persistent environmental pressures, plants have evolved sophisticated adaptive mechanisms centered on a conserved, multi-tiered signaling network encompassing kinase cascades, transcriptional reprogramming, and epigenetic regulation. This network detects environmental changes and activates downstream functional genes through regulatory cascades, forming complex stress response pathways (Riechmann and Meyerowitz, 1998; Jain and Chattopadhyay, 2013; Moore et al., 2014). This mechanism not only enables plants to respond to dynamically changing environments but also provides a crucial molecular framework for deciphering the evolutionary patterns of plant environmental adaptation.

The AP2/ERF transcription factors are central components of plant stress tolerance and growth regulation networks. Therefore, elucidating their molecular mechanisms and exploring their potential applications in breeding has become a major research focus. This study conducted a comprehensive bioinformatics analysis of the strong candidate genes within the AP2/ERF gene family, which are involved in responses to abiotic stresses such as drought, salinity, and cold, based on the whole-genome data of the alfalfa (*Medicago sativa*) cultivar ‘Xinjiang Daye’. Beyond expanding our functional understanding of the AP2/ERF gene family in alfalfa, this work identifies multiple key stress-responsive genes, providing valuable information on genetic resources for elucidating molecular mechanisms of stress tolerance in legumes to support molecular-assisted breeding efforts. These findings establish a solid theoretical basis for developing novel alfalfa varieties with enhanced resilience to abiotic stresses.

## Materials and methods

### Identification and sequence analysis of MsAP2/ERF genes

To identify the AP2/ERF gene family in alfalfa, this study utilized reference genomes from *Arabidopsis thaliana*, ‘Xinjiang Daye’ alfalfa and *Medicago truncatula* (obtained from https://modms.lzu.edu.cn/and https://phytozome-next.jgi.doe.gov/, respectively). Based on Hidden Markov Models (HMM), an initial screening was conducted using the PF00847 (AP2/ERF domain) feature spectrum. Subsequently, BLAST searches were performed on the alfalfa database using AP2/ERF domains from *Arabidopsis* and *Tribulus terrestris* proteins as query sequences. Subsequently, all candidate proteins were validated using the Pfam database (El-Gebali et al., 2019) and the NCBI-CDD database to ensure they contained complete AP2/ERF domains. After removing redundant sequences, the SMART online tool (https://smart.embl.de/) was further employed to eliminate sequences with incomplete AP2/ERF domains. Finally, the theoretical isoelectric point (pI) and molecular weight (MW) of the final non-redundant AP2/ERF proteins were calculated using the ExPASy online platform ().

### Phylogenetic analysis, gene structure identification and conserved motif distribution

A phylogenetic analysis was conducted on members of the AP2/ERF gene family from *M. sativa*, *A. thaliana*, and *M. truncatula*. Protein sequence data were obtained from the following sources: AP2/ERF protein sequences for *M. sativa* were retrieved from https://figshare; AP2/ERF protein sequences for *M. truncatula* were obtained from https://www.uniprot.org; *A. thaliana* AP2/ERF protein sequences were obtained from: https://modms.lzu.edu.cn. We constructed a phylogenetic tree for the three species using the ML method and default parameter values in MEGA 7, with 1000 bootstrap replicates, and visualized the phylogenetic tree using the ITOLS website (http://itol.itol.org/). Subsequently, phylogenetic analysis was performed on MsAP2/ERFs using the TBtools and MEME Suite 5.5.7 (https://meme-suite.org/meme/tools/meme) online programs to predict conserved AP2/ERF protein sequences (Liu et al., 2022). Finally, based on the alfalfa genome and gene annotation data, TBtools was employed to perform a visual analysis of the gene structure of the MsAP2/ERF genes.

### Chromosomal distribution, gene duplications, and evolutionary analysis

Based on the alfalfa genome annotation file, we employed the strategy proposed by Tian et al (Tian et al., 2020). to perform chromosome mapping analysis of AP2/ERF genes using TBtools software. Subsequently, genome-wide gene duplication events were detected using the MCScanX toolkit with default parameters. TBtools software was employed for comparative genomics between *M. sativa*, *A. thaliana*, and *M. truncatula*. Then, TBtools was used to perform collinearity analysis, using the Advanced Circos function to analyze collinearity among *Medicago sativa* gene duplication sequences. Finally, the ratios of the non-synonymous substitution rate (Ka) to the synonymous substitution rate (Ks) were calculated for each repeat gene pair using TBtools software to assess selection pressure.

### Cis-acting element analysis

Using the GTF/GFF3 sequence extraction module of TBtools software, potential promoter sequences spanning 2000 bp upstream of the CDS start site were obtained for members of the AP2/ERF gene family. The obtained sequences were analyzed for cis-acting elements using the PlantCARE database platform (https://bioinformatics.psb.ugent.be/webtools/plantcare/html/), systematically identifying core promoter elements and other regulatory features (Lescot et al., 2002). Finally, cis-elements were visualized using TBtools.

### RNA extraction and qRT-PCR analysis of 110 MsAP2/ERF gene homologs

To investigate the expression patterns of the 110 MsAP2/ERF genes under abiotic stress, transcriptome data of the ‘Zhongmu No. 1’ cultivar under cold, drought, and salt stress were analyzed based on the genome of the ‘Xinjiang Daye’ alfalfa. The transcriptome data were obtained from the MODMS database (https://modms.lzu.edu.cn/). The protein-coding sequences of the 110 MsAP2/ERF genes from the ‘Xinjiang Daye’ genome were extracted and used as queries in a BLASTP search against the unigene library assembled from the ‘Zhongmu No. 1’ transcriptome to identify homologous genes. The following criteria were applied for homology screening: E-value ≤ 1×10^-5^, percent identity ≥ 90%, and the highest-scoring target gene for each query was selected as the representative homolog. Expression levels of these homologs under each stress treatment were subsequently obtained.

Total RNA was extracted using the Total RNA Kit (OmegaBio-Tek, Norcross, Georgia, USA), then reverse transcribed into cDNA using the Prime ScriptTM RT Kit (TaKaRa, Dalian, Japan). Real-time quantitative PCR analysis was performed on an Applied Biosystems 7500 Real-Time Detection System (USA), utilizing SYBR Premix Ex Taq™ II pre-mixed reagent from Toyobo (Shanghai, China) in the reaction system. The amplification protocol followed the experimental parameters established by Shu et al (Shu et al., 2016), with three biological replicates set for each treatment. Using the MsActin gene as an internal control (Liu et al., 2016), quantitative primers for target genes were designed using Primer Premier 5 software (Premier Biosoft, USA). The specific primer sequences for the 15 MsAP2/ERF genes involved are detailed in **Supplementary Table S1**. The statistical significances of drought, salt, and cold stresses at five different time points were determined using one-way analysis of variance (ANOVA), followed by Tukey’s *post hoc* test for multiple comparisons.

### Plant materials and growth conditions

Alfalfa seeds were stratified at 4 °C on wet filter paper in Petri dishes for 48 hours, then germinated for 7 days in a 25 °C light incubator. The seedlings were divided into three groups: two groups were transplanted into a Hogland nutrient solution hydroponic system (with nutrient solution replaced every 7 days), while the other group was planted in a vermiculite-pumice mixture substrate (2:1, V/V). All groups were uniformly cultivated in an artificial climate chamber (light 16 h/dark 8 h, day/night temperature 25 °C/22 °C, humidity 60-70%). After 25 days of growth, hydroponic plants were treated with either 150 mmol·L^-^¹ NaCl or 20% PEG 6000, with leaf samples collected at 0, 3, 6, 12, and 24 h post-treatment. Soil-grown plants were transferred to a 2 °C cold environment for cold stress after 25 days of growth, with samples collected at 0, 1, 3, 5, and 7 days post-treatment. All leaf samples were rapidly frozen in liquid nitrogen and stored at -80 °C for future use.

## Results

### Identification of MsAP2/ERF family members

A phylogenetic analysis was conducted on members of the AP2/ERF gene family from *M. sativa, A. thaliana*, and *M. truncatula*. The protein sequences for *M. sativa, M. truncatula*, and *A. thaliana* are all sourced from https://modms.lzu.edu.cn. We constructed a phylogenetic tree for the three species using the ML method and default parameter values in MEGA 7, with 1000 bootstrap replicates, and visualized the phylogenetic tree using the ITOL website (iTOL: Interactive Tree Of Life). Subsequently, phylogenetic analysis was performed on MsAP2/ERFs using the TBtools and MEME Suite 5.5.7 (https://meme-suite.org/meme/tools/meme) online programs to predict conserved AP2/ERF protein sequences (Liu et al, 2022). Finally, based on the alfalfa genome and gene annotation data, TBtools was employed to perform a visual analysis of the gene structure of the MsAP2/ERF genes.

### Conservative motif distribution of MsAP2/ERFs family

**Figure 1:**
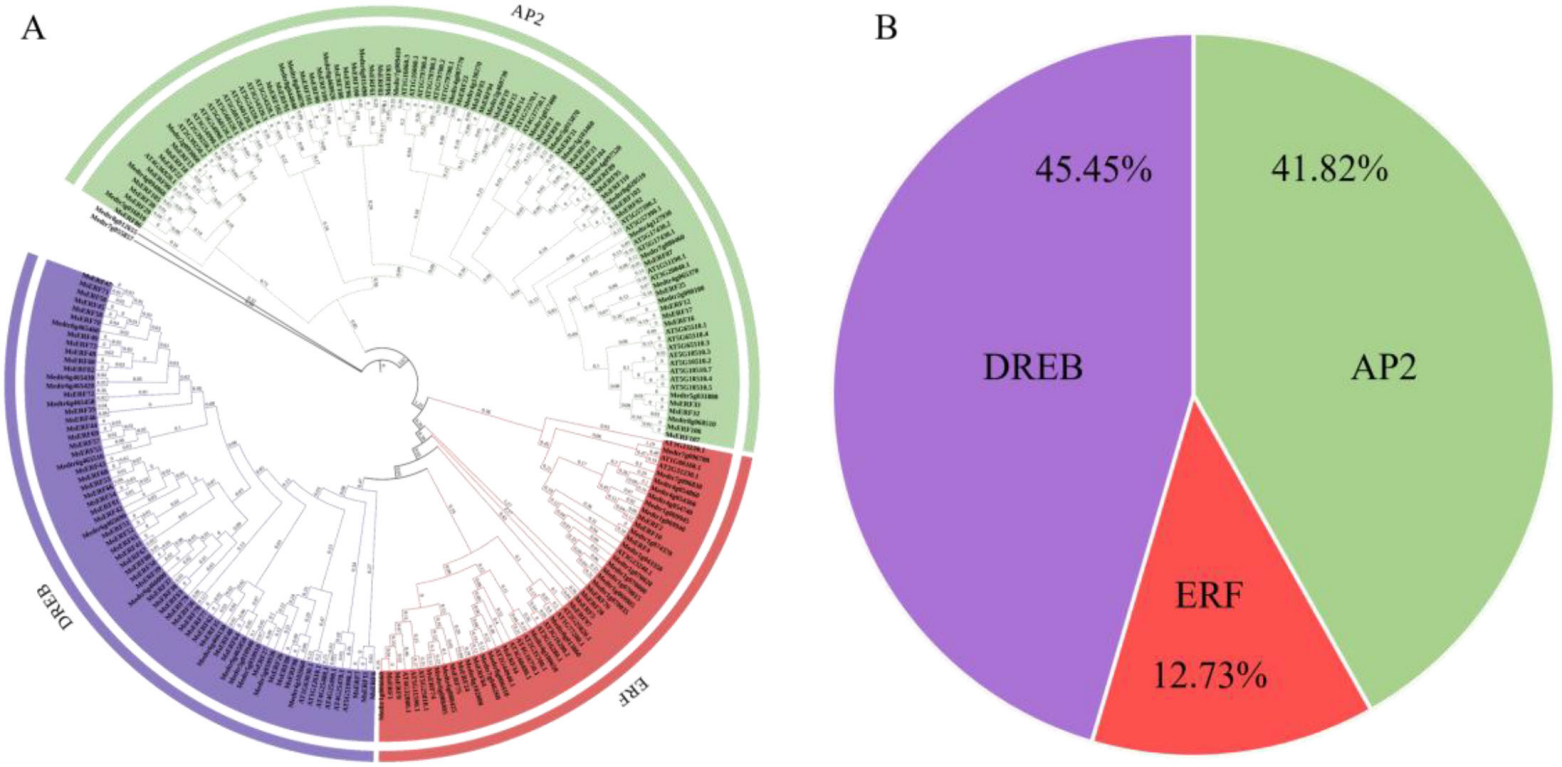
**(A)** Construction of phylogenetic tree based on AP2/ERF amino acid sequences of *M. sativa*, **(A)** *thaliana* and *M. truncatula*. **(B)** The proportion of AP2, ERF, and DREB subfamilies among 110 MsAP2/ERF genes.

Simultaneously, we conducted an in-depth analysis of conserved motifs and gene structures. Through homology searches and domain identification, we identified a total of 110 AP2/ERF genes. Based on the phylogenetic classification criteria established for the *Arabidopsis* AP2/ERF family, these genes were further categorized into three subfamilies: the DREB subfamily, comprising 50 genes; the ERF subfamily, with 14 genes; and the AP2 subfamily, which included the remaining 46 genes. These subfamilies represented 45.45%, 12.73%, and 41.82% of the total AP2/ERF genes, respectively. AP2 members were designated based on the presence of two tandem AP2/ERF domains (**Figures 1A, B**).

**Figure 2:**
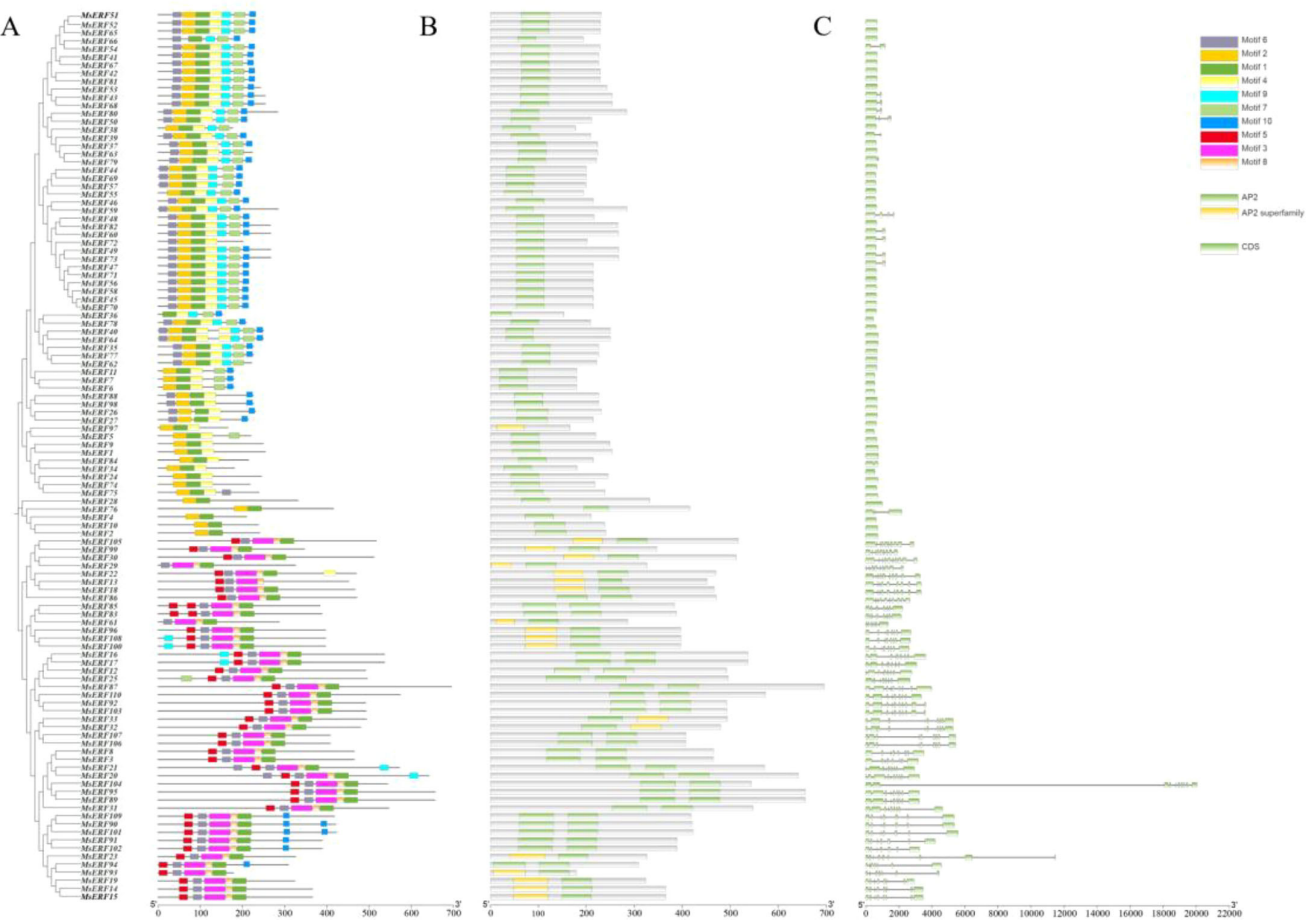
Phylogenetic relationship, motifs, and gene structure analysis of AP2/ERF genes in alfalfa. **(A)** Phylogenetic trees of 110 AP2/ERF genes in alfalfa. Conservative motif arrangement of MsAP2/ERF genes. Different colored boxes represent different motifs. The horizontal coordinate represents the CDS length of the gene. **(B, C)** The exon-intron organization of the MsAP2/ERF genes. The green box represents the exon; the black line represents the intron. The horizontal axis represents the full length of the gene.

As shown in **Figure 2**, approximately half of the MsAP2/ERF members lack introns. Among the remaining genes that contain introns, the number varies from 1 to 9. Of particular note is the identification of a gene (*MsERF104*) containing an intron region longer than 16,000 bp among the MsAP2/ERF family members.

### Chromosome distribution, duplication events and synteny analysis of MsAP2/ERFs

**Figure 3:**
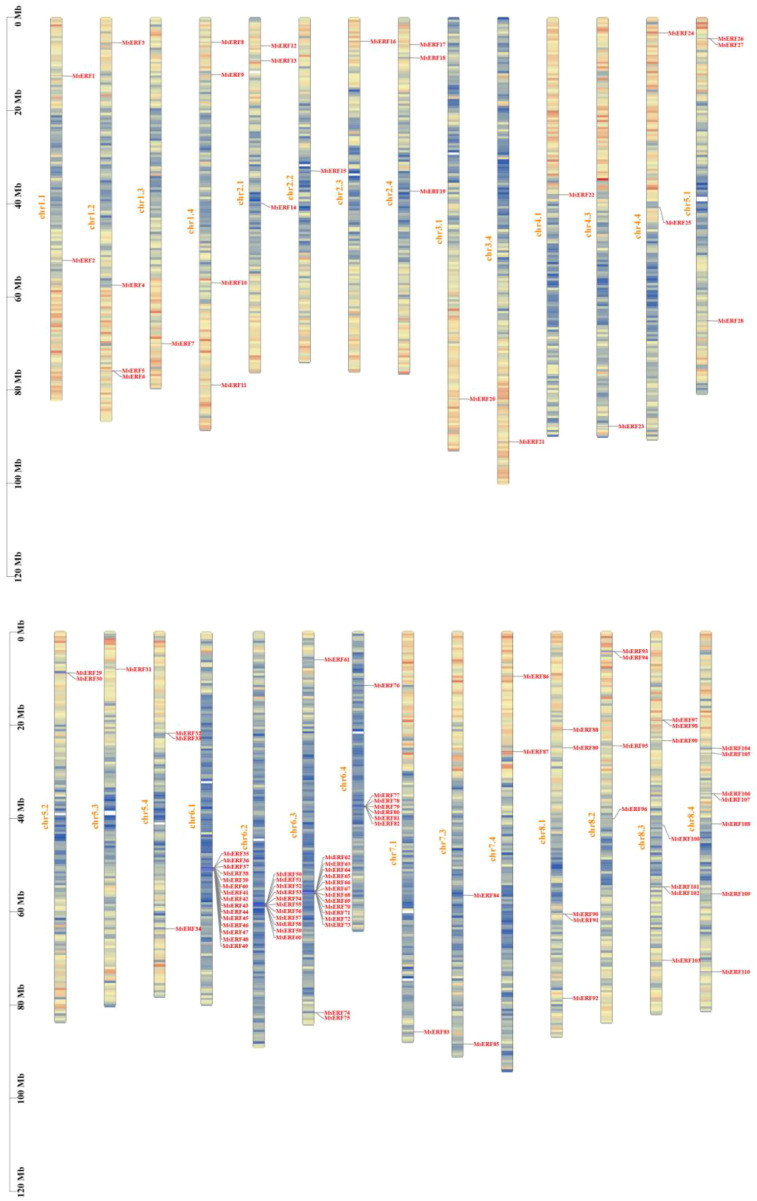
The distribution of MsAP2/ERF genes on alfalfa chromosomes. The colored lines represent each chromosome, and the black lines label the position of each MsAP2/ERF gene. The ordinate is the length of the chromosome.

Analysis of the chromosomal localization of the MsAP2/ERF genes, shown in **Figure 3**, indicates that the 110 MsAP2/ERF genes were distributed unevenly across 28 of the 32 chromosomes. Notably, no MsAP2/ERF genes were detected on chromosomes 3.2, 3.3, 4.2, or 7.2. Chromosome 6.1 harbors the highest number of genes, with a total of 15. By contrast, only one gene is located on each of the chromosomes 1.3, 2.2, 2.3, 3.4, 4.1, 4.3, 5.3, and 7.1, representing the chromosomes with the lowest gene densities. The MsAP2/ERF genes were named sequentially from *MsERF1* to *MsERF110* according to their physical positions on the chromosomes.

**Figure 4:**
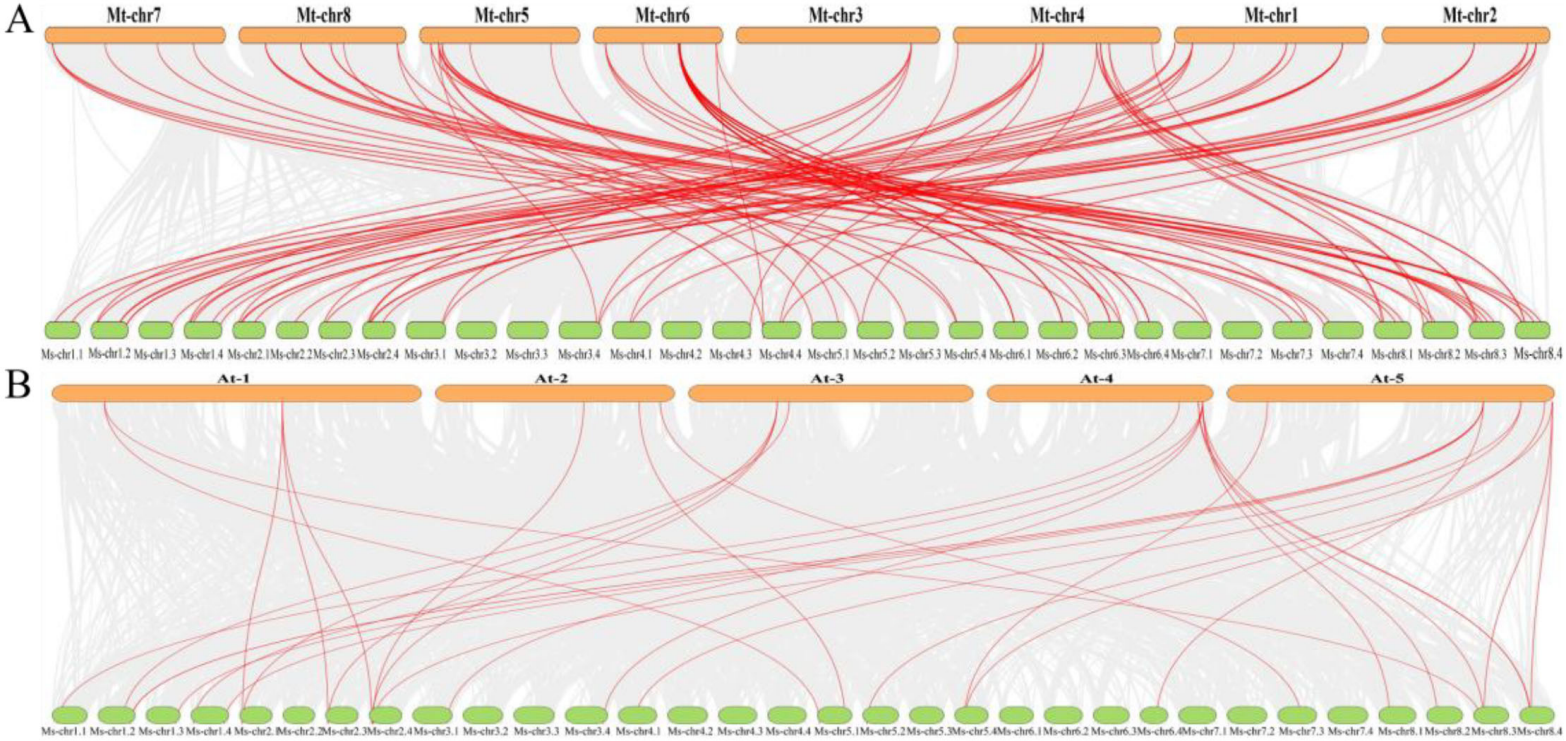
**(A)** Synteny analysis of AP2/ERFs between *M. sativa* and *M. truncatula*. **(B)** Synteny analysis of AP2/ERFs between *M. sativa* and *A. thaliana*. Red lines represent syntenic AP2/ERFs pairs.

**Figure 5:**
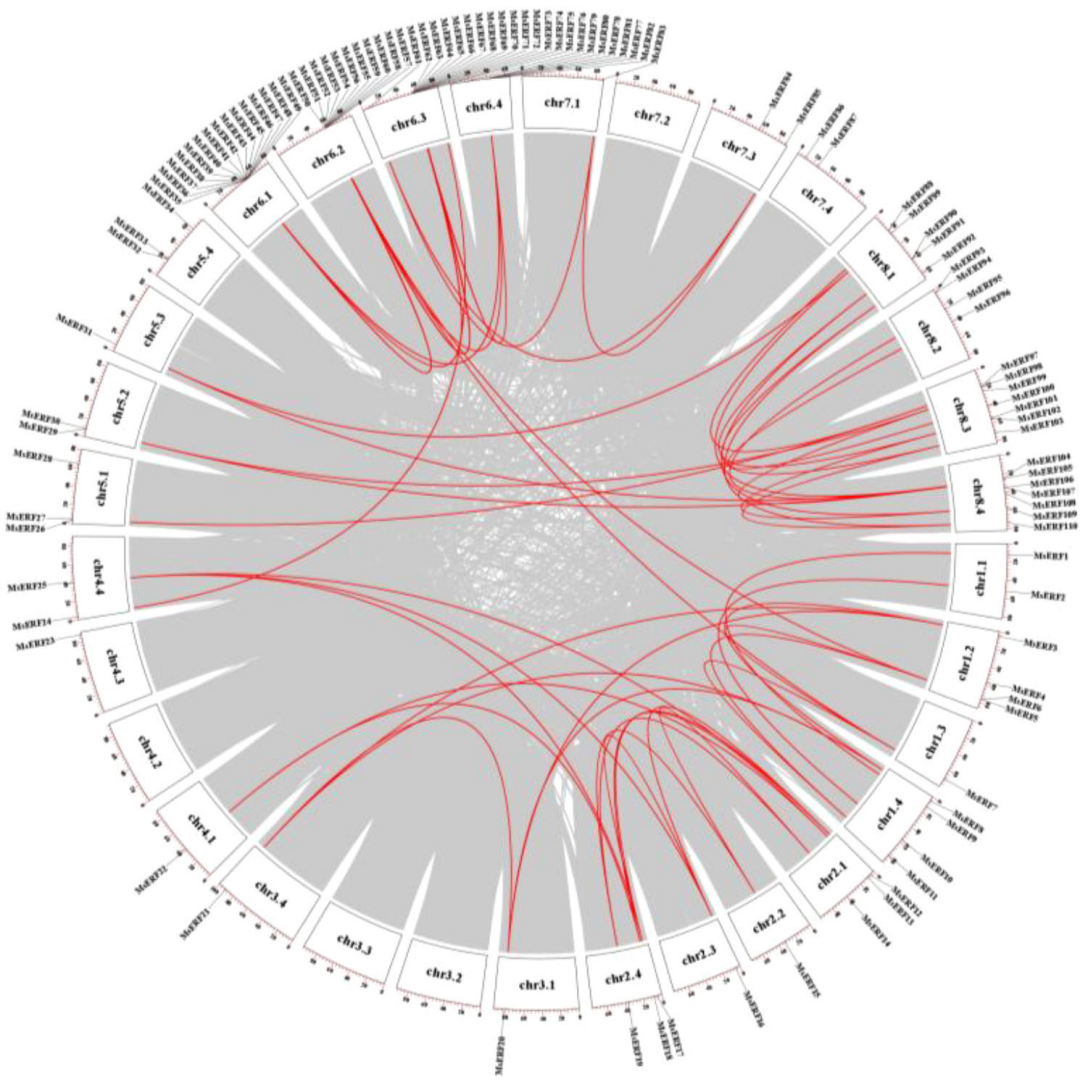
Collinearity analysis of MsAP2/ERF genes. The red line shows the MsAP2/ERF gene pairs replicated in alfalfa. The white boxes show the chromosomal position.

Collinearity analysis revealed 104 collinear gene pairs between alfalfa and *Tribulus terrestris*, and 33 with *Arabidopsis thaliana* (**Figure 4**). The Ka/Ks ratio, which measures the rate of non-synonymous (Ka) to synonymous (Ks) substitutions, serves as an indicator of selective pressure. All MsAP2/ERF genes exhibited Ka/Ks ratios substantially less than 1, indicating that they have undergone purifying selection (**Supplementary Table S4**). Repetition events among MsAP2/ERFs members were detected on chromosomes, with chromosomal regions containing two or more genes within 200 kb defined as tandem repetition events. A total of eight tandem duplication events were identified among the MsAP2/ERF genes. These include one pair on chromosome 1.2 (*MsERF32*/*MsERF33*), three pairs on chromosome 6.1 (*MsERF35*/*MsERF36*, *MsERF36*/*MsERF37*, *MsERF37*/*MsERF38*), three pairs on chromosome 6.4 (*MsERF55*/*MsERF56*, *MsERF58*/*MsERF59*, *MsERF59*/*MsERF60*), and one pair on chromosome 6.4 (*MsERF79*/*MsERF80*) (**Figure 5**).

### Analysis of cis-acting elements in MsAP2/ERF promoters

**Figure 6:**
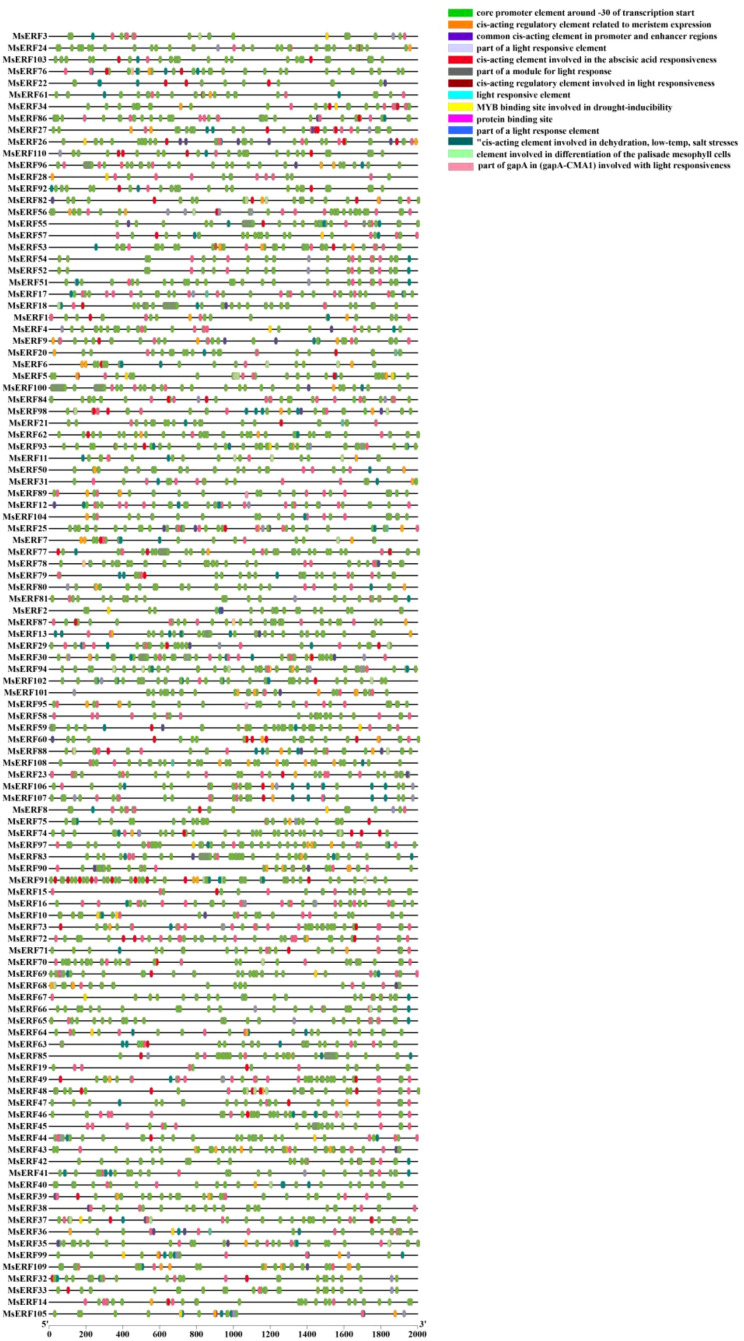
Analysis of the 2000 bp promoter regulatory elements of the MsAP2/ERF genes. Colored blocks represent different types of cis-regulatory elements and their relative positions within each MsAP2/ERF gene promoter region.

To gain a deeper understanding of the functions of the MsAP2/ERF genes family in alfalfa, this study systematically analyzed cis-acting elements within the promoter regions (approximately 2000 bp) of 110 MsAP2/ERF genes using the PlantCARE database (**Figure 6**). Analysis results indicate that the vast majority of cis-elements contained within the promoter regions of MsAP2/ERF genes are closely associated with their functions, primarily involving plant maturation processes, plant hormone responses, and responses to biotic/abiotic stresses.

Further functional classification statistics were conducted on the 14 major cis-acting element types identified. The results indicate that for non-biotic stress responses, two element types (primarily DRE/MBS) were directly associated with responses to drought, dehydration, low temperature, and salt stress. Notably, 103 MsAP2/ERF genes (representing 93.6% of the analyzed genes) harbored these elements within their promoter regions, underscoring the pivotal involvement of this gene family in the regulation of abiotic stress responses. For plant hormone responses, one cis-acting element type, primarily ABRE, was significantly associated with abscisic acid (ABA) response and was detected in the promoters of 62 MsAP2/ERF genes. Notably, for light responses, six cis-acting element types related to light signaling were identified, exhibiting the greatest diversity among all functional categories of elements. The category ‘Regulation of Plant Growth and Development’ encompasses two primary types, with 59 gene promoters containing elements associated with cell differentiation and 18 gene promoters containing elements linked to meristem activity.

In summary, the promoter regions of MsAP2/ERF genes contain a wide array of regulatory elements, among which non-biotic stress-related elements (notably DRE/MBS) and ABA-responsive elements (ABRE) are the most abundant. This composition offers a critical molecular foundation for the involvement of these genes in complex stress adaptation and hormone-mediated signaling. Additionally, the prevalence of light-responsive and growth/development-related elements further implies a broad functional repertoire of this gene family.

### Expression analysis of homologous genes of MsAP2/ERF genes under abiotic stress

**Figure 7:**
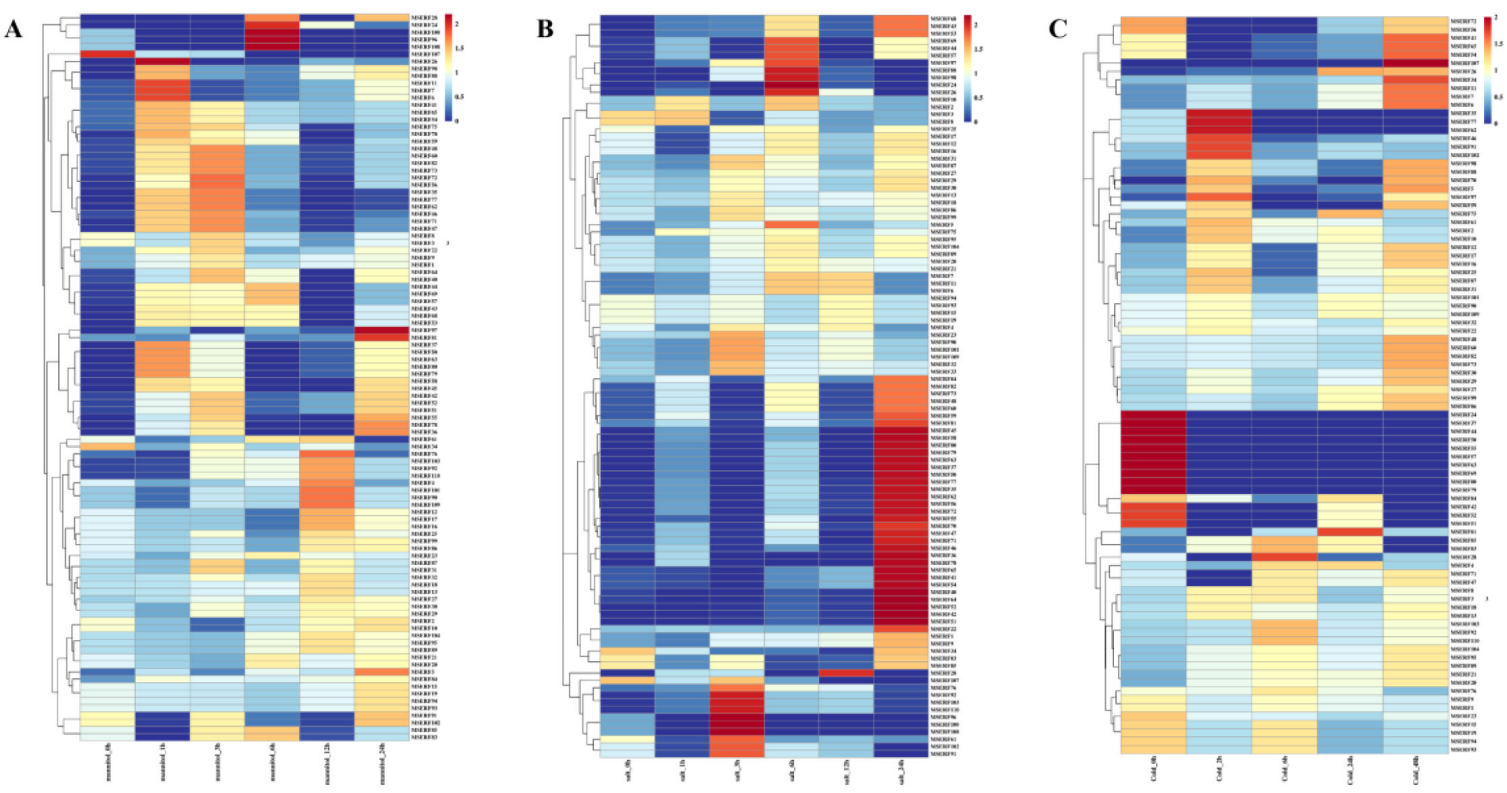
Expression profiles of MsAP2/ERF genes under various treatments. **(A–C)** Heatmaps representing RNA-seq expression levels of MsAP2/ERF genes, with blue and red corresponding to low and high expression, respectively.

Analysis revealed that among genes with elevated 24-hour response levels, 38, 25, and 14 genes exhibited significant upregulation under salt, low-temperature, and drought stress, respectively. Among these, the 12 genes *MsERF36*, *MsERF41*, *MsERF42*, *MsERF45*, *MsERF51*, *MsERF52*, *MsERF54*, *MsERF55*, *MsERF58*, *MsERF65*, *MsERF78*, and *MsERF81* collectively participate in responses to all three types of stress (**Figures 7A–C**).

To validate the expression patterns of these 12 core MsAP2/ERF genes under these three types of stress, RT-qPCR analysis was performed on alfalfa leaves. Under salt stress, all 12 genes were expressed, but exhibited distinct temporal dynamics. *MsERF55*, *MsERF65*, and *MsERF78* showed downregulation during the first 0–12 hours of stress exposure, but were significantly upregulated and reached peak expression at 24 hours. By contrast, *MsERF41*, *MsERF45*, and *MsERF52* were upregulated during the initial 0–12 hours and peaked at 12 hours, but were significantly downregulated at 24 hours. Under drought stress, except for *MsERF58*, the remaining 11 MsAP2/ERF genes were significantly upregulated in leaves, with expression levels peaking at 24 hours, indicating that members of this family primarily exert a positive regulatory role during drought stress.

**Figure 8:**
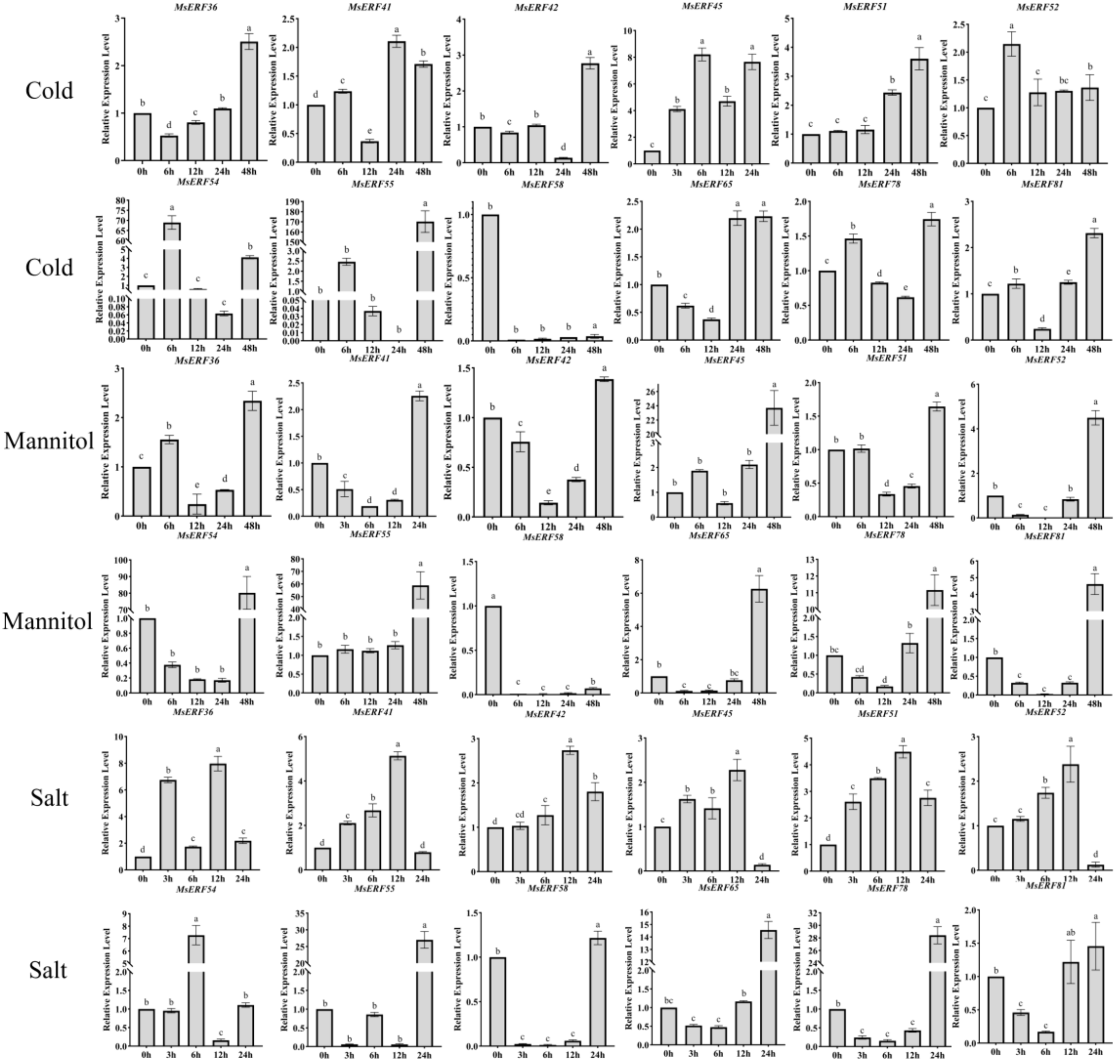
Expression patterns of 12 selected MsAP2/ERF genes in response to abiotic stresses-cold, salt, and drought. Different lowercase letters indicate significant differences in response time under the same stress condition (P < 0.05).

Under low-temperature treatment, gene expression patterns were more diverse: *MsERF54* and *MsERF52* rapidly upregulated to peak levels at 6 h, then gradually declined; *MsERF45* exhibited a fluctuating pattern of initial increase, subsequent decrease, and final rise; *MsERF36*, *MsERF42*, *MsERF51*, and *MsERF55* were up-regulated to peak levels at 48 h; while *MsERF58* remained undetectable throughout the process. This indicates that most MsAP2/ERFs genes in leaves exhibit differentiated regulatory responses to low temperatures (**Figure 8**).

## Discussion

The AP2/ERF superfamily is one of the most ubiquitous and critical transcription factor families in plants. It plays a central role in modulating key developmental transitions, including seed germination, floral development, leaf senescence, and fruit ripening, as well as in orchestrating transcriptional reprogramming in response to abiotic stresses (e.g., salinity, drought, and cold) and biotic stresses (e.g., pathogen infection) (Schmidt et al., 2013; Zhu et al., 2014). The AP2/ERF superfamily represents a major and highly diverse group of plant transcription factors. Comparative genomics approaches have enabled genome-wide identification and functional characterization of AP2/ERF members across a wide range of plant species (Faraji et al., 2020; Zeng et al., 2023). However, research on the AP2/ERF superfamily in alfalfa remains limited, and the functional roles and mechanistic underpinnings of its members in abiotic stress responses have not been fully elucidated.

This study aimed to elucidate the evolutionary relationships within the MsAP2/ERF genes family by constructing a cross-species phylogenetic tree and analyzing gene sequences. The MsAP2/ERF genes family is divided into three highly conserved subfamilies: DREB, ERF, and AP2. Shu et al (Shu et al., 2016). identified four subfamilies within the AP2/ERF gene family of *M. truncatula*, including the RAV subfamily in addition to DREB, ERF, and AP2. RAV simultaneously contains dual conserved domains AP2 and B3, participating in plant development, stress response, and hormone signal transduction by regulating the expression of downstream target genes. The RAV subfamily has few members in plants, and its presence has not been detected in alfalfa. This may be because the RAV subfamily in alfalfa exhibits atypical domains. In alfalfa, the number of AP2/ERF genes is slightly lower than in *Arabidopsis* (147), soybean (147), and *Medicago truncatula* (123). This is primarily due to the relatively lower number of members in the DREB and ERF subfamilies compared to 98 members *A. thaliana* and *M. truncatula* (Shu et al., 2016), 122 in *Arabidopsis* (Feng et al., 2005), and 120 in soybean (Wang et al., 2022).

Analysis of protein physicochemical properties revealed differences in relative molecular mass, amino acid composition, and isoelectric point (pI) between MsAP2/ERFs, indicating that functional variations of AP2/ERF proteins under different microenvironmental conditions are similar to those observed in other plant proteins (Feng et al., 2017; Li et al., 2020). Differences in isoelectric points affect their binding affinity and specificity to DNA (Riechmann et al., 2000). These proteins exhibit diversity in amino acid content, molecular weight, and isoelectric point, potentially reflecting their specific functions in different biological processes.

Gene duplication serves as a crucial mechanism driving the evolution and functional diversification of gene families, playing a pivotal role in plants’ adaptation to environmental changes and developmental processes (Bowers et al., 2003). For instance, in Arabidopsis and rice, approximately 15-20% of genes originate from tandem duplications, and these genes are essential for evolution, disease resistance, and responses to abiotic stresses (Abdullah et al., 2021; Musavizadeh et al., 2021). Through chromosome mapping and gene structure analysis, this study revealed gene duplication events occurring during genome expansion and evolution, which generated genes with conserved structures via negative selection (Zhu et al., 2014). This study identified 110 MsAP2/ERF members in alfalfa and discovered eight pairs of tandem repeats among them. This finding aligns with conclusions from other dicotyledonous plants (Kavas et al., 2015; Yu et al., 2022), further confirming the universality and importance of segmental duplication in the evolution of this gene family. Chromosomal distribution analysis indicated that these 110 genes are distributed across 28 chromosomes in alfalfa. By comparison, 123 members are distributed across the 8 chromosomes of *M. truncatula* (Shu et al., 2016). This difference may be related to their genome ploidy levels. *M. truncatula* is diploid, and may better preserve the organizational structure of the AP2/ERF family within legume genomes. By contrast, *Medicago sativa* is an autotetraploid, and gene dispersion reduces the risk of complete functional loss of the gene family due to localized chromosomal variation. It also provides more opportunities for generating new regulatory combinations or protein interaction variations through mechanisms such as non-allelic homologous recombination, potentially promoting functional diversification of this family in a tetraploid background. In other legumes, 301 members are contained within the 20 chromosomes of soybean, and 540 members are distributed across the 28 chromosomes of red clover (Wang et al., 2022; Liu et al., 2023). These findings indicate that the AP2/ERF gene family typically exhibits polychromosomal distribution in plants, potentially reflecting functional diversity and specificity that supports adaptation to complex environments and developmental regulation.

In this study, the MsAP2/ERF genes pair exhibited an average Ka/Ks ratio below 1, indicating that these MsAP2/ERFs underwent strong purifying selection during their evolutionary history (**Supplementary Table S4**). These findings align with prior reports from genome-wide analyses of the AP2/ERF superfamily across various plant species (Fu, 2024; Tang et al., 2025). Furthermore, collinearity analysis revealed that MsAP2/ERFs exhibit strong collinear relationships with MtERF in the dicotyledonous plant *M. truncatula*, with 104 colinear gene pairs, whereas only 33 colinear gene pairs were identified with *A. thaliana*. indicating that the number of collinear gene pairs among AP2/ERF genes in *M. truncatula* and *M. sativa* is greater than that observed between *M. truncatula* and *A. thaliana*.

In *Arabidopsis*, a total of 50 conserved motifs outside the AP2/ERF domain have been identified. The evolution of numerous gene families is primarily determined by the organization of gene structures (Xu et al., 2012; Xiao et al., 2017). MsAP2/ERFs proteins contained 10 distinct conserved motifs (with motif 6 distributed across 98 gene members), and all 110 members contained at least one motif. The coding sequence lengths of these genes exhibited significant variation, reflecting the structural complexity of this family within the genome. *MsERF104* contains an intron exceeding 16,000 bp in length, which significantly exceeds the typical length range for genes in this family. Long introns are not frequently encountered in the homologous genes of *A. thaliana* or *M. truncatula*. The presence of such extended intronic regions may accommodate a greater diversity of cis-regulatory elements, potentially enabling the development of a more layered and highly coordinated gene expression regulatory network. Meanwhile, most MsAP2/ERF genes exhibit structural features characterized by 1–2 introns or no introns, collectively indicating that the AP2 domain is highly conserved within this family. These findings are consistent with those reported in *Arabidopsis*, maize, and cotton (Sakuma et al., 2002; Zhang et al., 2022). The high conservation of intron number and position within each subfamily strongly supports the phylogenetic classification of MsAP2/ERF genes and reflects functional conservation across distinct subgroups.

Cis-acting element, as non-coding DNA sequences located upstream of genes, play a crucial role in plant responses to abiotic stresses such as drought, high salinity, and low temperatures, by binding specifically to transcription factors (Tang et al., 2025). The transcriptional activity of genes is largely determined by the types and combinations of cis-acting elements contained within their promoter regions (Yamaguchi-Shinozaki and Shinozaki, 2005). Among these, members of the ERF subfamily primarily bind to the GCC-box, participating in ethylene response and biotic stress-related regulation (Gu et al., 2000), while the DREB subfamily activates the expression of genes associated with cold and dehydration stress by recognizing the CRT/DRE element (Fujimoto et al., 2000). Promoter analysis revealed that these gene promoters not only contain multiple hormone response elements (e.g., ABA, SA, and IAA) but also harbor drought and cold stress response elements in several members. Collectively, these findings suggest that MsAP2/ERFs genes may exert core regulatory functions in alfalfa growth, development, and stress adaptation by integrating hormone signals with environmental stress signals (Yang et al., 2020).

Transcriptomics, by analyzing gene expression profiles at specific developmental stages, can reveal tissue-specific expression patterns of genes, thereby providing crucial insights into the functions of individual genes. This technology has been widely applied across multiple fields including basic science, agriculture, and medicine (Shu et al., 2016). Transcriptomic analyses of *Tribulus terrestris* under multiple stress conditions have revealed global gene expression patterns and identified the AP2/ERF transcription factor family (Zhang et al., 2023). Although the reference genomes and transcriptome data were derived from two distinct cultivated varieties, ‘Xinjiang Daye’ and ‘Zhongmu No. 1’, stringent homology alignment criteria (E-value < 1e-5, Query cover > 90%) ensured that gene identification and expression analysis were based on highly conserved homologs with clear inter-varietal correspondence. This approach effectively minimized the potential impact of inter-varietal polymorphism on result interpretation. Consequently, the focus of this study was not on comparing varietal differences, but on systematically deciphering the overall expression patterns of the alfalfa AP2/ERF family in response to multiple abiotic stresses and identifying key members within this family. Based on publicly available transcriptome data, we identified 12 genes that were differentially expressed under salt, drought, and low-temperature stress. Our findings indicate that the MsAP2/ERF gene family may play a significant role in responses to salt and drought stress, providing key directional guidance for further gene mining and functional analysis.

Research has confirmed the regulatory role of AP2/ERFs gene in plant responses to abiotic stress (Licausi et al., 2013). Expression analysis of 12 MsAP2/ERF genes was performed under three abiotic stress conditions (drought, salinity, and cold) over a series of time courses. The results indicate that the expression levels of various genes exhibit significant differences under different stress conditions. Eight MsAP2/ERF genes were significantly upregulated under salt stress conditions, a finding consistent with previous observations regarding AP2/ERF genes in cauliflower (Li et al., 2017). Drought stress treatment induced the expression of 11 MsAP2/ERF genes. Furthermore, previous studies have reported the presence of drought-induced AP2/ERFs gene in *Pinus massoniana*, suggesting that this gene family may possess a conserved regulatory function in plant drought responses (Sun et al., 2022). Cold-induced AP2/ERF genes have also been reported in various plant species, including walnut (Zhao et al., 2022) and Siberian apricot (Zhang et al., 2024).

Notably, *MsERF45*, *MsERF54*, and *MsERF55* all responded to the three types of stress. This may be related to tandem repeats, which drive changes in regulatory elements and protein structures of relevant genes. These adaptations enable them to coordinate responses to multiple stress signals, thereby conferring broader stress tolerance. Tandem repeats promote an increase in the number of AP2/ERF family members, allowing certain genes to exert regulatory functions under combined stress conditions. In addition, *MsERF45* showed significant induction under salt and low-temperature stress; After 24 h of drought treatment, the expression levels of *MsERF45*, *MsERF52*, *MsERF54*, and *MsERF55* were up-regulated by 3-6-fold (**Figure 8**). RT-qPCR results further confirmed that both salt and drought treatments significantly induced the expression of *MsERF45*, *MsERF54*, and *MsERF55*. These findings suggest that *MsERF45*, *MsERF54*, and *MsERF55* may be involved in processes related to plant responses to abiotic stress and warrant further investigation.

## Conclusion

This study conducted a systematic and comprehensive bioinformatics analysis of the MsAP2/ERF genes family in alfalfa. A total of 110 MsAP2/ERF genes were identified, unevenly distributed across 28 chromosomes. Phylogenetic analysis classified them into three highly conserved subfamilies: ERF, DREB, and AP2. The promoter regions are rich in various stress response elements. Transcriptome and qRT-PCR results further indicate that multiple genes (such as *MsERF45*, *MsERF54*, *MsERF55*) are significantly expressed under salt, drought, and low-temperature stress.

A limitation of this study is that the specific biological functions of these genes in stress resistance have not been functionally validated. Future efforts should prioritize *in vivo* functional studies, utilizing techniques such as genetic transformation and gene editing, to further elucidate their roles and molecular regulatory mechanisms. Such work will provide valuable information on genetic resources and a theoretical basis for improving stress resistance in alfalfa through molecular breeding.

## Data Availability

The data supporting this research are publicly available in the MODMS database (Alfalfa Multi-omics Database, URL: https://modms.lzu.edu.cn/). Genome sequences, gene models, and annotation files used for identifying and analyzing the MsAP2/ERF gene family were derived from data resources provided by this platform. These resources are based on the reference genome described by Fang et al. (2024). All relevant data generated or analyzed in this study are included in this article and its supplementary information files.
